# Asthma Management in Sickle Cell Disease

**DOI:** 10.1155/2013/604140

**Published:** 2013-11-10

**Authors:** Esteban Gomez, Claudia R. Morris

**Affiliations:** ^1^Department of Hematology-Oncology, Children's Hospital & Research Center Oakland, Oakland, CA 94609, USA; ^2^Division of Pediatric Emergency Medicine, Department of Pediatrics, Emory University School of Medicine, 1645 Tullie Circle, NE, Atlanta, GA 30329, USA

## Abstract

Asthma is a common comorbid factor in sickle cell disease (SCD). However, the incidence of asthma in SCD is much higher than expected compared to rates in the general population. Whether “asthma” in SCD is purely related to genetic and environmental factors or rather is the consequence of the underlying hemolytic and inflammatory state is a topic of recent debate. Regardless of the etiology, hypoxemia induced by bronchoconstriction and inflammation associated with asthma exacerbations will contribute to a cycle of sickling and subsequent complications of SCD. Recent studies confirm that asthma predisposes to complications of SCD such as pain crises, acute chest syndrome, and stroke and is associated with increased mortality. Early recognition and aggressive standard of care management of asthma may prevent serious pulmonary complications and reduce mortality. However, data regarding the management of asthma in SCD is very limited. Clinical trials are needed to evaluate the effectiveness of current asthma therapy in patients with SCD and coincident asthma, while mechanistic studies are needed to delineate the underlying pathophysiology.

## 1. Introduction

The association between sickle cell disease (SCD) and asthma has been described in numerous studies. An increased prevalence of asthma in patients with SCD has also been documented as have increased morbidity and mortality amongst patients with coincident SCD and asthma. The pathophysiology underlying the relationship between asthma and SCD has become a topic of interest, although little is known. Further insight will hopefully lead to targeted interventions that can help minimize the complications associated with coincident asthma and SCD. For now, asthma management based on the guidelines published by the National Institutes of Health (NIH) should be implemented to minimize morbidity and mortality for patients with SCD and asthma. Concerns regarding the use of typical treatments for asthma in patients with SCD have arisen, but the benefits of optimal asthma treatment outweigh the risks of possible side effects.

## 2. Prevalence of SCD and Asthma

The NIH estimates that SCD affects from 90,000 to 100,000 Americans. One out of 500 African-American births and 1 out of 36,000 Hispanic-American births are estimated to result in patients affected with SCD [1]. Meanwhile, the Center for Disease Control has published survey data documenting increased rates of asthma diagnoses in 2009 compared to 2001. Importantly, the greatest rise in asthma rates over the same time period was in African-American children. The prevalence of asthma in African-American children in 2009 throughout the United States was noted to be 17%, whereas in 2001 it was noted to be approximately 9% [2]. These numbers contrast with estimates of asthma in African-American children with SCD. A study of an infant cohort from the Cooperative Study of Sickle Cell Disease (CSSCD) found a prevalence of 16.8% amongst those African-American children; these children were enrolled between 1978 and 1988 when the NIH estimates of asthma prevalence were even lower for children and for African Americans (3.4–5.8%) [3, 4]. In California, asthma rates for African-American children are 20% [5], while a retrospective review of patients screened by pulmonary function testing at the Northern California Comprehensive Sickle Cell Center suggested the presence of obstructive disease (alone or in combination with restrictive disease) in 58% of the 124 adults and children screened by standard pulmonary function testing (PFTs) [6, 7]. This is consistent with other studies of lower airway obstruction and airway hyperreactivity (AHR) in children with sickle cell disease. Koumbourlis et al. completed a cross-sectional study that found lower airway obstruction in 35% of children with SCD [8]. When they evaluated AHR in the same study population, 54% had a positive bronchodilator response as measured by pulmonary function testing. Ozbek et al. evaluated AHR with methacholine challenge testing and found that 77% of children with SCD had a positive test result [9]. Multiple other studies have found a prevalence of AHR by one method or another in children with SCD that has fallen within the range of 35–77% [7, 10–16]. This is in comparison to the reported 20% prevalence for AHR in the general pediatric population [17]. Together, these studies document that SCD patients have a much higher prevalence of AHR than would otherwise be expected. This in turn implies that the prevalence of asthma or an “asthma-like” condition amongst patients with SCD is higher than would have been expected if AHR and SCD did not have an underlying relationship.

## 3. Clinical Consequences of Coincident Asthma and SCD

An increase in morbidity and mortality in patients with both asthma and SCD has been noted in multiple studies. A recent study by Knight-Madden et al. found an increased ten-year mortality in SCD patients experiencing “current” asthma with a hazard ratio of 11.2 [18]. Increased rates of acute chest syndrome (ACS), stroke, and vaso-occlusive episodes (VOE) have all been documented in patients with asthma and SCD [3, 14, 16, 18–26]. Glassberg and colleagues found that a past history of asthma was associated with an increased risk of emergency department utilization for both pain and ACS in children with SCD [25]. Boyd et al. found that SCD patients with a diagnosis of asthma were 4 times as likely to develop ACS after admission for VOE when compared to SCD patients without asthma [19]. Nordness et al. performed a retrospective chart review from over a 5-year span at a single center and found that children with SCD and asthma had an increased incidence of ACS and stroke compared to children with SCD alone [21]. Knight-Madden et al. performed a retrospective study of children with SCD at the largest sickle cell clinic in Jamaica and found an increased incidence of recurrent ACS in children with SCD and asthma compared to children with SCD alone [20]. Boyd et al. studied an infant cohort from the CSSCD and found that children with SCD and a clinical diagnosis of asthma had approximately twice as many episodes of ACS and nearly three times as many pain crises [3]. Boyd et al. also performed a single-center retrospective study in children with sickle cell disease with abnormal pulmonary function tests and those with normal lung function studies. They found that children with lower airway obstruction had twice the risk for a pain crisis or ACS compared to children with normal lung function [23]. Boyd et al. also performed a prospective study including 1,963 children and adults with SCD and found a more than twofold increased risk of mortality for patients with asthma, independent of other established risk factors for mortality [22]. Consistent with this observation, Nouraie and colleagues recently reported a hazards ratio of death of 5.8 for history of asthma in nearly 500 children and adolescences prospectively enrolled in the Pulmonary Hypertension and the Hypoxic Response in Sickle Cell Disease (PUSH) study who were followed over a median of 3 years [27]. Case reports of asthma-related deaths have also been described [28]. Strong evidence is accumulating from multiple investigators and centers that asthma is an independent risk factor for early mortality in both adults and children with SCD [15, 18, 22, 27, 29], yet no randomized controlled trials of asthma therapies in SCD have been published to date [30]. Since asthma is a modifiable risk factor, effective asthma management may ultimately impact morbidity and mortality in SCD.

## 4. Unique and Overlapping Pathophysiology in Asthma and SCD

A number of inflammatory pathways contribute to asthma [31–34], many of which are outside the scope of this review. Interestingly, several well-documented mechanisms of asthma are known to contribute to the pathophysiology of SCD. These specific pathways provide a framework to guide additional investigation into the commonly found asthma-like condition in children with SCD.

One potential underlying connection between SCD and asthma is the inflammation seen with each disease process. SCD is a proinflammatory state with baseline elevations of cytokines such as interferon-*γ*, tumor necrosis factor-*α*, and others [35]. Vascular adhesion molecule-1 is also known to be elevated in SCD [36]. During VOEs, these inflammatory molecules have been seen to rise even higher [35, 36]. This inflammation could in turn result in a propensity towards clinical asthma. Supporting this hypothesis is the documentation of increased mortality and an enhanced inflammatory response to allergens in mice with SCD and experimentally induced asthma [37].

 Leukotrienes are a particularly intriguing commonality between asthma and SCD. They are key signaling molecules involved in both inflammation and bronchoconstriction. Recent studies have shown a potential role for leukotrienes in the pathogenesis of both VOE and ACS [38–40]. 5-Lipoxygenase converts arachidonic acid (AA) into leukotrienes. AA is made from secretory phospholipase A2 (sPLA2), an enzyme known to be increased in patients with SCD at baseline and during VOEs and ACS episodes [41]. In one study, RBC transfusion was found to prevent ACS predicted by elevated sPLA2 compared to the 63% of patients with an elevated sPLA2 randomized to standard care (no transfusion) who went on to develop ACS [42]. There is also data to suggest a role for elevated sPLA2 in asthma in general [43], which is of interest given the difficulty in clinically differentiating ACS from asthma in a patient with SCD. Thus, these molecules have a theoretical link from SCD to asthma. Although controlled clinical trials have not yet been done, montelukast, an inhibitor of leukotriene activity, is an intriguing candidate for the treatment of asthma in patients with SCD [44].

Dysregulated arginine metabolism and excess arginase activity has also been implicated in the pathophysiology of both asthma [29, 45–51] and SCD [7, 29, 52–56]. Elevated plasma levels of the arginine analog asymmetric dimethyl arginine (ADMA) also contribute to arginine dysregulation in both asthma [57, 58] and SCD [59–61]. L-Arginine is the obligate substrate for both the arginases and nitric oxide synthetases (NOS). When acted on by NOS, L-arginine is converted into nitric oxide (NO) and citrulline. NO is a potent vasodilator that plays a role in maintaining bronchodilatory tone. NO in exhaled breath (eNO) is also an established marker of chronic inflammation in asthmatics [62]. Mixed results on eNO levels in SCD have been reported. Low eNO levels in SCD patients with asthma and ACS were initially reported in contrast to asthmatic patients without SCD [63, 64]. However, a more recent study demonstrated elevated eNO levels in nonatopic children with SCD compared to normal controls [65], again illustrating a variance in NO metabolism in SCD versus control subjects. A plausible explanation may be found by considering potential mechanisms that contribute to the clinical phenotype that differed in each study. Metabolic profiling has revealed an arginine deficiency among subgroups of patients with SCD that is associated with clinical phenotypes and a mortality risk that varies from steady-state to acute events [46, 47, 52, 53]. Simultaneous plasma arginine and eNO levels were not measured in any of these studies, a variable that may impact eNO concentration [66].

**Figure 1:**
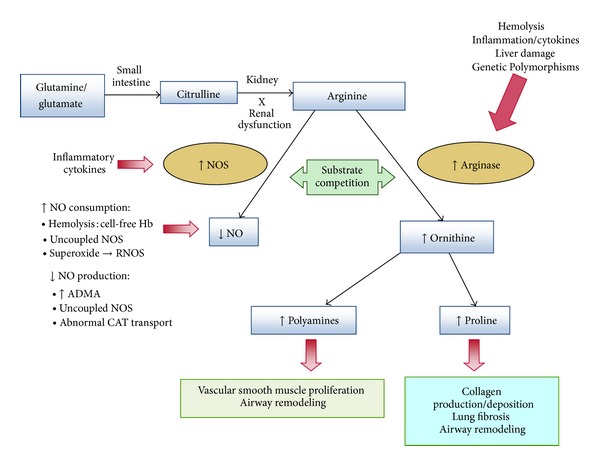
Altered arginine metabolism in sickle cell disease (SCD). Dietary glutamine and glutamate serve as a precursor for the *de novo* production of arginine through the citrulline-arginine pathway. Arginine is synthesized endogenously from citrulline primarily via the intestinal-renal axis [80]. Arginase and nitric oxide synthase (NOS) compete for arginine, their common substrate. Asymmetric dimethyl arginine (ADMA) is an arginine analog and NOS inhibitor that is elevated in SCD [59–61]. In SCD, bioavailability of arginine and nitric oxide (NO) is decreased by several mechanisms linked to hemolysis [53, 54, 81, 82]. The release of erythrocyte arginase during hemolysis increases plasma arginase levels and shifts arginine metabolism towards ornithine production, limiting the amount of substrate available for NO production [53]. The bioavailability of arginine is further diminished by increased ornithine levels because ornithine and arginine compete for the same transporter system for cellular uptake (cationic amino acid transporter—CAT) [83, 84]. Despite an increase in NOS, NO bioavailability is low due to low substrate availability [53], NO scavenging by cell-free hemoglobin released during hemolysis [85], and through reactions with free radicals such as superoxide and other reactive NO species [86–88]. Superoxide is elevated in SCD due to low superoxide dismutase activity [89, 90], high xanthine oxidase activity [87], and potentially as a result of uncoupled NOS [91, 92] in an environment of low arginine and/or tetrahydrobiopterin concentration or insufficient NADPH. Superoxide will react with NO to form reactive nitric oxide species (RNOS) including peroxynitrite [93], which can contribute further to cell damage and cell death. Endothelial dysfunction resulting from NO depletion and increased levels of the downstream products of ornithine metabolism (polyamines and proline) likely contributes to the pathogenesis of lung injury, pulmonary hypertension, and asthma in SCD. This model has implications for all hemolytic processes. This new disease paradigm is now recognized as an important mechanism in the pathophysiology of SCD. Reproduced/modified with permission from the American Society of Hematology [81].

Arginase metabolism of L-arginine decreases its bioavailability to NOS, effectively limiting NO production [46] and contributes to AHR in asthma [48, 49, 67, 68]. In addition, downstream byproducts of arginase activity (proline and polyamines) are associated with collagen deposition, airway remodeling, smooth muscle proliferation, and fibrosis [69, 70], potentially contributing to both obstructive and restrictive lung disease (Figure 1) [29, 56, 71]. Although this is only one of many mechanisms contributing to AHR in asthma, this is an intriguing mechanism worthy of further study given the abnormal arginine metabolome in SCD [52, 53, 56].

 Adults with SCD are arginine deficient, [53, 72] while children tend to have normal levels at steady-state compared to control subjects [52]. However both adults and children experience an acute decrease in plasma arginine concentrations during VOEs and episodes of ACS [52, 73]. Of interest, parenteral arginine significantly decreased total opioid use and improved pain scores in children with SCD hospitalized for VOE compared to placebo in a recently published randomized, placebo-controlled trial [74]. Plasma arginase activity is elevated in SCD as a consequence of inflammation, liver dysfunction, and, most significantly, by the release of erythrocyte arginase during intravascular hemolysis [53], which has been demonstrated by the strong correlation between plasma arginase levels and cell-free hemoglobin levels [53] and other markers of increased hemolytic rate including LDH [53, 75]. In a recent prospective cohort study, Field et al. [13] reported no relationship of methacholine-induced AHR with asthma symptoms or physician diagnosis of asthma. However, AHR was noted to be related to increased LDH levels supporting hemolysis as a potential mechanism [75] contributing to airway hyper-reactivity and asthma in SCD [29]. In contrast, Pritchard et al. found no association between LDH and AHR in a mouse model of SCD. Given that murine erythrocytes contain minimal arginase, this model is unfortunately limited to evaluate the impact of hemolysis-driven increases in arginase levels as a mechanism of AHR. However, this study did find that SCD alone in the mouse model induces a baseline lung pathology that increases large and small airway resistance and primes the lungs for increased inflammation and AHR after allergic sensitization [76]. Hagar and colleagues [14] found an association of asthma in children with SCD who had an elevated tricuspid regurgitant jet velocity on Doppler echocardiography, a measure that is strongly associated with hemolytic rate [77]. Of interest, this association of asthma and pulmonary hypertension risk was not found in adults with SCD [14]. Future studies are needed to validate this hypothesis that includes asthma [7, 29] in the hemolytic sub-phenotype of SCD [78], particularly in lieu of the controversy surrounding the hyperhemolysis pathway [79].

## 5. Asthma Diagnosis in Patients with SCD

The diagnosis of asthma for patients in SCD is complicated by an overlap between the symptoms and findings of asthma exacerbations and episodes of ACS. According to the national asthma education and prevention program (NAEPP) expert panel report (EPR), the working definition of asthma is as follows: asthma is a chronic inflammatory disorder of the airways in which many cells and cellular elements play a role: in particular, mast cells, eosinophils, T lymphocytes, macrophages, neutrophils, and epithelial cells. In susceptible individuals, this inflammation causes recurrent episodes of wheezing, breathlessness, chest tightness, and coughing, particularly at night or in the early morning. These episodes are usually associated with widespread but variable airflow obstruction that is often reversible either spontaneously or with treatment. The inflammation also causes an associated increase in the existing bronchial hyperresponsiveness to a variety of stimuli. Reversibility of airflow limitation may be incomplete in some patients with asthma [94]. Meanwhile, ACS is defined by the finding of a new infiltrate on CXR with at least one of the symptoms of fever, cough, sputum production, and dyspnea [95]. Although the recurrent nature of asthma symptoms associated with a trigger may help distinguish these two entities, ACS can also be recurrent and may present with wheezing, tachypnea, and rhinorrhea [95, 96]. Symptoms may also improve with beta-agonists [95], a common asthma therapy. A CXR infiltrate is required to diagnose ACS, but asthma exacerbations can be triggered by pulmonary infections with associated CXR abnormalities. These common characteristics may make it difficult to definitively distinguish between the two entities, and the safest management for some cases may be treatment for both possibilities.

Asthma is a clinical diagnosis. However, at this time, it is not clear if clinical features of asthma are similar in patients with SCD compared to the general population. Phillips et al. reported a familial pattern of inheritance of asthma among first degree relatives of probands with SCD and asthma [97]. Field and colleagues found a sibling history of asthma as a risk factor for pain crisis in children with SCD, suggesting disease-modifying effects of asthma due to familial factors commonly seen in typical asthma [98]. Since parental history of asthma was not associated with significantly increased risk of pain or ACS episodes in this study, it is not clear if the association seen with sibling history was purely genetic, due to environmental influences or both. Interestingly, a more recent study by Field et al. [13] reported no relationship of methacholine-induced AHR with typical asthma symptoms or physician diagnosis of asthma, which suggests an “asthma-like” condition in SCD that differs from atopic asthma [7, 29]. Regardless, patient history should be reviewed for symptoms suggestive of recurrent AHR or airflow obstruction to help establish a diagnosis of asthma, especially given the high prevalence of atopic asthma in African Americans. Typical symptoms include wheezing, coughing, difficulty breathing, or chest tightness. It is also important to review the patient history for possible triggers and for the timing of symptoms. Classic triggers include exercise, respiratory infections, and inhaled allergens. In particular, the role of environmental tobacco smoke exposure should not be discounted when assessing patients with repeated respiratory exacerbations/wheezing problems, since this is a modifiable risk factor that could improve morbidity. Asthmatic symptoms are typically worse at night or upon awakening. The patient should also be questioned about risk factors for asthma such as a personal or family history of allergic rhinitis, asthma, and eczema. Asthma symptoms are frequently missed by clinicians unless the information is specifically elicited in careful questioning about the presence of such symptoms like nighttime cough and poor exercise capacity [7], as families often will not spontaneously offer this information without prompting. 

A history of snoring, daytime somnolence, presence of tonsillar, and adenoidal hypertrophy should prompt clinician to obtain an overnight polysomnogram to assess for nocturnal hypoxemia and sleep disordered breathing. Sinusitis [99], obstructive sleep apnea [100], and gastroesophageal reflux [101] are common comorbidities found with asthma that trigger asthma symptoms and need be addressed when present in order to obtain optimal asthma control. In addition, control of environmental factors and avoiding known asthma triggers is critical to minimize exacerbations. 

 Abnormal lung function is commonly seen in SCD. Obstructive changes are typical in young children [7, 16, 102, 103], whereas a restrictive pattern dominates in older children and adults with SCD [16, 104]. MacLean et al. showed that there is a significant decline in spirometric lung volumes across childhood in SCD. The average decline for FEV1 and total lung capacity is 2.93 and 2.15% predicted/year for males and 2.95 and 2.43% predicted/year for females, which is similar to children with cystic fibrosis and more than those with typical asthma [105]. Early evidence suggests some protection from decline in lung function in children with SCD after successful bone marrow transplant [106].

 Although spirometry can provide objective evidence of persistent asthma by demonstrating reversible airflow obstruction, normal spirometry does not exclude an asthma diagnosis. In practice, the diagnosis is made based on patient history and clinical symptoms. Asthma is a reversible condition and spirometry often normalizes at baseline after recovery from an acute exacerbation. When the diagnostic criteria for asthma are not demonstrated with spirometry, bronchoprovocation can objectively demonstrate AHR to support a diagnosis of asthma. Conversely, a negative test would help exclude a diagnosis of asthma. Methacholine challenge (MCh) has been demonstrated to be the most sensitive test for AHR in patients with SCD [9, 12, 13]. While this test has been used safely for decades in patients with SCD, a recent case report describing a pain crisis following a MCh underscores the need to use caution and implement this test only in patients with the appropriate risk-benefit ratio [107].

## 6. Asthma Management in Sickle Cell Disease

 Unfortunately, controlled trials of asthma therapy in SCD have not yet been done. In the absence of any evidence-based clinical guidelines, it is recommended that asthma in SCD be managed based on established NAEPP guidelines for the treatment of asthma (as shown in the list below) [7, 94]. As with all guidelines, it is imperative to weigh the risks and benefits for each specific patient prior to their implementation; the expertise of a pulmonologist as well as a hematologist will help with these decisions.

*Sickle Cell Disease Asthma Management [7]*

1. Treat asthma based on NIH asthma guidelines [108–113]: inhaled bronchodilators as rescue medication for respiratory symptoms (nebulized albuterol, +/− ipratropium bromide) and corticosteroids for moderate/severe exacerbations. Utilize oral prednisone with slow taper at 1-2 mg/kg/day. A 5-day burst may be insufficient and a slower taper over 2 weeks may be indicated. Case reports of rebound pain and acute chest syndrome have been described after corticosteroids are withdrawn [114–118]; however, they should not be withheld during an asthma exacerbation. Use of steroids in SCD is a topic of current interest and investigation; however, no interventional studies of SCD and asthma have been performed to date. Pulmonologists familiar with SCD universally recommend treating asthma per NIH guidelines in SCD.
2. Liberal use of inhaled steroids (asthma controller medication) as first line for persistent asthma symptoms; consider other controller medications like leukotriene inhibitors (Montelukast).
3. Consult pulmonary or hematology specialist when placing SCD patient on corticosteroids.
4. Hospital admission for all asthma exacerbations requiring corticosteroids.
5. Low threshold to admit mild asthma exacerbations given associated complications.
6. Close monitoring and followup are essential.
7. Pulmonary function testing as an outpatient should be followed annually.
8. Screen SCD patients with asthma symptoms for pulmonary hypertension by Doppler echocardiography annually.

## 7. Controversies in Asthma Management for SCD

### 7.1. Oxygen Use

 Although counterintuitive, the safety of oxygen use for patients with SCD during asthma exacerbations has been questioned. A case series on three patients with SCD found that continuous oxygen over several days could cause a reduction in the production of new sickled cells. However, after cessation of oxygen therapy, there was a compensatory increase in the production of sickled cells [119]. It is important to note that this study was performed in patients who were not otherwise hypoxic and so should not be applied to patients with SCD and hypoxemia in the midst of an asthmatic exacerbation or possible ACS. Furthermore, it is well established that hypoxia can promote cell sickling and so needs to be reversed in patients with SCD. Although patients with SCD and no respiratory impairment do not warrant oxygen therapy, use of short-term oxygen in the acute setting of an asthma exacerbation should never be discouraged.

### 7.2. *β*_2_-Agonists

*β*_2_-Agonists have a role in both the acute and chronic management of asthma. Their primary action is to promote smooth muscle relaxation, leading to bronchodilation. Short-acting *β*_2_-agonists are a mainstay in the treatment of acute asthma exacerbations. Long-acting *β*_2_-agonists are recommended for the control and prevention of symptoms for asthmatics with moderate or severe-persistent asthma. However, research has raised concern regarding the use of *β*_2_-agonists in the setting of sickle cell disease.

Multiple studies have shown that stimulation of *β*_2_-adrenergic receptors on red and white blood cells promotes cellular adhesion [120–125]. Theoretically, this would suggest that the use of *β*_2_-agonists in sickle cell patients could lead to an increased risk for VOEs. However, this theoretical concern has never been demonstrated clinically.

The use of long-acting *β*_2_-agonists (LABA) in African-American patients is particularly controversial given the findings of the Salmeterol Multicenter Asthma Research Trial (SMART). This study raised concern regarding the safety profile of LABAs specifically for African Americans [126]. However, subsequent study on the use of LABAs in combination with inhaled corticosteroids as specifically recommended in the current NIH guidelines has shown a clinical benefit for African Americans without any increased risk of harm [127].

 Also, concerning with regards to *β*_2_-agonists is the increased incidence of prolonged QTc interval documented in patients with SCD [128]. Independent of sickle cell disease, the use of *β*_2_-agonists in patients with prolonged QTc has been associated with an increased risk for life-threatening cardiac events [129]. This combination of observations suggests that it may be prudent to perform a baseline ECG in patients with SCD prior to initiating therapy with *β*_2_-agonists. However, it is important to note that a specific clinical harm from the use *β*_2_-agonists in SCD patients has not been found. In contrast, the clinical benefit of *β*_2_-agonists in the management of sickle cell patients with asthma is well established.

### 7.3. Corticosteroids

 Airway inflammation is a major contributor to the pathophysiology of acute and chronic asthma. Corticosteroids reduce AHR, inhibit cell migration and activation, and halt allergic reactions, all of which are helpful in the management of asthma. Inhaled corticosteroids (IHCs) are the most effective medication to date for the management of persistent asthma with minimal systemic effects and have been shown to reduce the severity and incidence of asthma exacerbations. In acute exacerbations, systemic corticosteroids shorten the course of symptoms and help prevent rebound asthma symptoms. However, significant concern regarding the use of corticosteroids in patients with SCD exists. Chronic corticosteroid usage is associated with an increased risk of avascular necrosis, and entity certain SCD patients are already prone to develop [130]. Corticosteroids also cause leukocytosis, a known risk factor for vaso-occlusive episodes in SCD [131]. Data regarding the specific use of systemic corticosteroids in the treatment SCD patients with acute asthma exacerbations is lacking, while studies in ACS have been performed. Of concern, retrospective studies and case reports have found that the use of systemic corticosteroids is associated with an increased risk for rebound pain crises in patients with SCD [114, 132]. In a retrospective study examining 5,247 hospitalizations across 32 hospitals for ACS in over 3,000 individuals with SCD, significant variability of clinical practice utilizing corticosteroids was noted. Corticosteroids were associated with an increased length of stay, and a higher 3-day readmission rate [132]. However, it is likely that in this uncontrolled setting where use of corticosteroids is not standard practice, sicker patients may have received corticosteroids. In addition, only 48% of patients with an asthma diagnosis received corticosteroid treatment [132]. Since asthma requiring hospitalization typically qualifies as a moderate-to-severe exacerbation, standard practice would almost universally include use of corticosteroids for a non-SCD patient with asthma and significant respiratory symptoms. A relative risk of 3.2 for readmission of patients with asthma compared to those without asthma was also noted [132]. It is not possible to determine whether this reflected more severe ACS in patients with asthma or undertreatment of asthma with corticosteroids. There are also studies documenting the benefits of dexamethasone for pain and acute chest syndrome [133–138] and others that demonstrate that corticosteroids can be safely used in SCD patients without adverse events [134, 139, 140]. It is more likely that the potent anti-inflammatory effects of these agents target inflammation leading to clinical improvement; however, abrupt withdrawal triggers rebound inflammation in some patients. Therefore, a longer course with a slow taper may be necessary to minimize risks, although controlled trials are lacking. The debate around rebound pain has led to reluctance by clinicians to treat these patients with corticosteroids, while mounting evidence demonstrates that the risks of asthma itself outweigh those of steroid use. Pulmonologists familiar with SCD universally recommend treating asthma per NIH guidelines in SCD. Systemic corticosteroid bursts should be provided for treatment of moderate-to-severe asthma exacerbations. Some clinicians recommend that these bursts should include 1-2-week tapers, while other clinicians prefer shorter courses to address the risk for rebound pain potentially associated with the use of corticosteroids. With either approach, patients should be monitored closely after corticosteroid use for any evidence of rebound pain. Also, SCD patients with persistent asthma should be treated with daily inhaled-corticosteroid therapy to minimize asthma-related morbidity.

### 7.4. Other Therapies

 Leukotriene receptor antagonists (LTRA), like Montelukast, may be considered as an adjuvant therapy. Leukotriene inhibitors may be a promising therapy in SCD given evidence of elevated urinary levels of LTE4 at baseline and during pain crises or ACS [39]. Studies evaluating the efficacy of leukotriene inhibitors in decreasing morbidity in patients with SCD are needed. 

Recent studies linking acetaminophen use with asthma risk and severity [141, 142] suddenly create a new controversy in the treatment of SCD. Since chronic acetaminophen use is a mainstay of pain management, acetaminophen use may have a unique risk profile in SCD not previously considered. Clinical studies are warranted in this population to evaluate the impact of this common analgesic on glutathione redox [143, 144], hemolysis, and asthma risk in SCD. 

 Since many complications of SCD result in part from an increased hemolytic rate [78], it is logical to think that decreasing hemolysis would improve morbidity and mortality in SCD. Chronic blood transfusions reduce the rate of hemolysis and decrease painful VOE and ACS episodes [145] and may indirectly reduce progression of lung disease, although this association has not yet been studied. Similarly, hydroxyurea has been shown to reduce painful episodes and ACS events [146] and possibly improve chronic hypoxemia [147], thereby reducing pulmonary morbidity in SCD, although its impact specifically on asthma has not been studied. Novel therapies that target improvement in arginine and NO bioavailability may also have a role in the treatment of pulmonary and vascular complications of SCD. The impact of bone marrow transplant on pulmonary dysfunction deserves further consideration [106]. 

## 8. Conclusion

 The relationship between asthma and SCD is a complex one. The high frequency of asthma in this population cannot be attributed to genetic predisposition alone and likely reflects in part the contribution of overlapping mechanisms shared between these otherwise distinct disorders. Patients with SCD may potentially be at risk for an asthma-like condition triggered or worsened by hemolysis-driven release of erythrocyte arginase, low nitric oxide bioavailability, and subsequent alterations in the arginine metabolome in addition to classic familial asthma. Much has been learned about the pathophysiology connecting asthma and SCD, but more research is needed to identify safe and effective treatment options. Early recognition and aggressive standard of care management of asthma may prevent serious pulmonary complications. The national asthma education and prevention program (NAEPP) asthma management guidelines [94] should be implemented with the assistance of a hematologist and likely a pulmonologist. Certain risks specific to patients with SCD need to be considered when treating their asthma, but these need to be weighed against the undeniable benefits of effective asthma management.
